# History Shaped the Geographic Distribution of Genomic Admixture on the Island of Puerto Rico

**DOI:** 10.1371/journal.pone.0016513

**Published:** 2011-01-31

**Authors:** Marc Via, Christopher R. Gignoux, Lindsey A. Roth, Laura Fejerman, Joshua Galanter, Shweta Choudhry, Gladys Toro-Labrador, Jorge Viera-Vera, Taras K. Oleksyk, Kenneth Beckman, Elad Ziv, Neil Risch, Esteban González Burchard, Juan Carlos Martínez-Cruzado

**Affiliations:** 1 Department of Medicine, University of California San Francisco, San Francisco, California, United States of America; 2 Institute for Human Genetics, University of California, San Francisco, California, United States of America; 3 Helen Diller Family Comprehensive Cancer Center, University of California San Francisco, San Francisco, California, United States of America; 4 Department of Urology, University of California San Francisco, San Francisco, California, United States of America; 5 Department of Biology, University of Puerto Rico, Mayagüez, Puerto Rico; 6 Department of Biology, University of Puerto Rico, Río Piedras, Puerto Rico; 7 Department of Genetics, Cell Biology & Developmental Biology, University of Minnesota, Minneapolis, Minnesota, United States of America; 8 Division of Research, Kaiser Permanente, Oakland, California, United States of America; 9 Department of Bioengineering and Therapeutic Sciences, University of California San Francisco, San Francisco, California, United States of America; State University of New York College at Oneonta, United States of America

## Abstract

Contemporary genetic variation among Latin Americans human groups reflects population migrations shaped by complex historical, social and economic factors. Consequently, admixture patterns may vary by geographic regions ranging from countries to neighborhoods. We examined the geographic variation of admixture across the island of Puerto Rico and the degree to which it could be explained by historic and social events. We analyzed a census-based sample of 642 Puerto Rican individuals that were genotyped for 93 ancestry informative markers (AIMs) to estimate African, European and Native American ancestry. Socioeconomic status (SES) data and geographic location were obtained for each individual. There was significant geographic variation of ancestry across the island. In particular, African ancestry demonstrated a decreasing East to West gradient that was partially explained by historical factors linked to the colonial sugar plantation system. SES also demonstrated a parallel decreasing cline from East to West. However, at a local level, SES and African ancestry were negatively correlated. European ancestry was strongly negatively correlated with African ancestry and therefore showed patterns complementary to African ancestry. By contrast, Native American ancestry showed little variation across the island and across individuals and appears to have played little social role historically. The observed geographic distributions of SES and genetic variation relate to historical social events and mating patterns, and have substantial implications for the design of studies in the recently admixed Puerto Rican population. More generally, our results demonstrate the importance of incorporating social and geographic data with genetics when studying contemporary admixed populations.

## Introduction

Worldwide patterns of modern human genetic variation have been shaped by a long history of demographic events, such as migrations or changes in population size. Population geneticists have always recognized the role of geography in the distribution of human genetic variation [1], [2] and have substantiated observations with historical and archeological sources [3], [4]. Geographic components have been included in the study of the original settlement of human populations or in the detection of ancient demographic events, such as Neolithic expansions [5], and population structure, even at fine scales [6], [7], [8].

However, most of these studies focus on questions of human evolution dating back several millennia, and not on more recent events, namely the mass migrations that have occurred in post-Columbian times. For example, admixture in Latin America is the result of demographic processes involving groups from three continents that took place since the arrival of Columbus in 1492. The observed heterogeneity in the relative proportions of African, European, and Native American ancestry among contemporary populations across the Americas is the reflection of local variables such as the pre-Colonial size of the existing indigenous populations and the relative importance of the African slave trade [9], [10], [11]. The differences in genetic admixture among individuals within a population result from assortative mating based on socioeconomic status and skin color [12], the consequences of a social hierarchy established by the colonial powers. Despite this heterogeneity, most research efforts on admixture have analyzed populations as a whole while largely ignoring the spatial and social factors that have shaped contemporary admixture patterns [13].

Puerto Rico is an ideal venue to understand the impact of historical and social factors on modern genetic patterns. The island has a higher degree of tri-hybrid admixture than most countries in Latin America [14]. The number of indigenous Taínos living in Puerto Rico at the moment of the first contact with Europeans has been estimated at 110,000 [15]. From that moment, Spanish settlers, mostly single men, began mating with Taíno women. Through war, slavery and disease, the Taíno population was drastically reduced. Consequently, African slaves were introduced as a source of labor. Like most of the Caribbean, the colonial economy in Puerto Rico was largely based on the sugar trade to Europe and other markets, and slave plantations were concentrated in the important sugar-producing areas. In turn, manufactured products from these areas were used to purchase new slaves in different parts of Africa and in other American colonies [16], [17]. Today, almost all modern Puerto Ricans are admixed descendents of the three ancestral populations (Taínos, Europeans, and Africans). However, current social perceptions and administrative classifications fail to capture this complexity: in the 2000 U.S. census only 4.2% of Puerto Ricans self-identified as “two or more races”, and 95.8% self-categorized into a single “race”, including “white” (80.5%), “black or African American” (8.0%), “some other race” (6.8%) and “American Indian or Alaskan Native” (0.4%) [18]. 98.8% considered themselves Hispanic or Latino.

Variation in ancestry within a population has social implications and has often been the basis for discrimination and socioeconomic differences across Latin America [19]. Dominance by European descendents has been inherited from colonial times, resulting in lower wages and less education for people with African or Native American appearance [13], [20], [21]. These social differences also impact health and disease risk. In Puerto Rico, we have described complex interactions between social factors, genetic ancestry and risk for disease [22]. Genetic ancestry confers opposite risks for asthma among Puerto Ricans in low versus high socioeconomic groups. The relative importance of genetic ancestry and social factors in health and disease is still controversial, and likely involves interactions between both elements [23], [24]. Thus, the examination of the interaction between genetic ancestry and social factors is imperative.

Here, we assess the genetic admixture components of a census-based sample of Puerto Ricans using a set of ancestry informative markers (AIMs). We used GIS to integrate this admixture information with information available for geography, socioeconomic status, and historical elements associated with the colonial sugar economy and the slave trade. We characterized the spatial patterns of genetic and SES variation across Puerto Rico and investigated the historical factors that shaped them. To our knowledge, this investigation represents the first attempt to integrate comprehensive information from population genetics, historical sources, socioeconomic status and geography at a local level.

## Results

### Genetic structure

**Figure 1:**
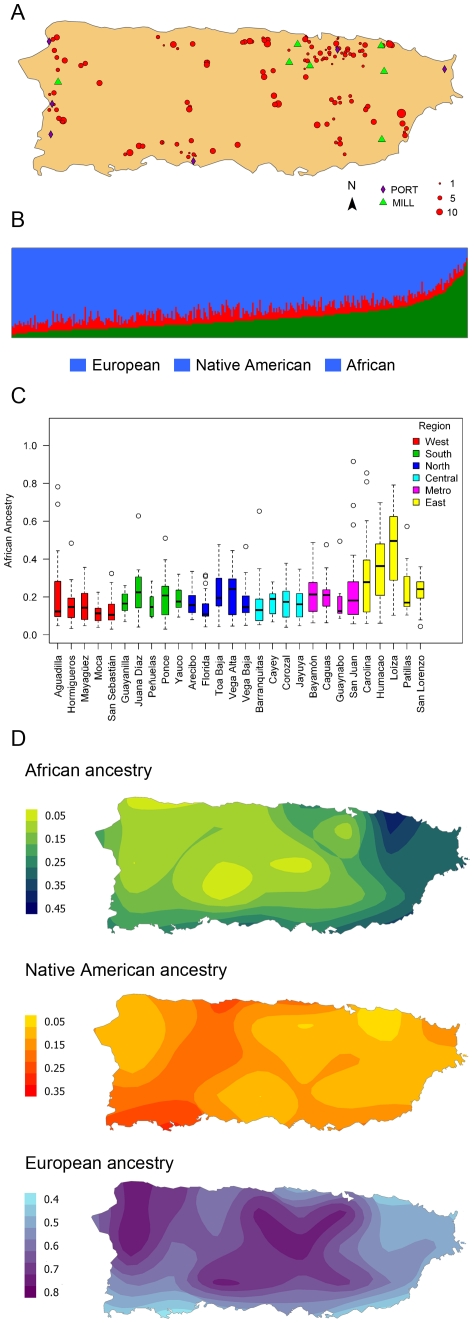
Distribution of individuals and their ancestry estimates. (A) Distribution of samples across the island. Symbols are proportional to the number of samples included for each census block. Location of sugar mills and ports is also included. (B) Ancestry estimates for each individual are shown as a thin vertical line partitioned into different colored components representing inferred membership in the ancestral groups. (C) Comparisons of African ancestry between municipalities, grouped by region. (D) Interpolation plots showing the geographical distribution of ancestry.

We applied the Bayesian clustering algorithm STRUCTURE [25], [26] using an ancestral reference set of Native Americans, Africans and Europeans to estimate admixture proportions in our census-based the Puerto Rican sample (Figure 1A). Average ancestry values for the Puerto Rican population were 15.2% (±7.2), 21.2% (±14.4), and 63.7% (±15.2) for the Native American, African, and European contributions, respectively (Table 1). As previously shown for other Latin American populations, extensive variation among individuals was observed (Figure 1B) especially in the European and African components. Overall, these ancestral components showed greater variances than the Native American component.

**Table 1:** Admixture estimates and SES per region.

		Native American		African		European		SES		
Region	N	Mean	SD	Mean	SD	Mean	SD	Mean	SD	Weight
Central	87	14.3	6.1	16.9	9.1	68.8	10.2	2.31	0.85	0.111
East	137	13.5	7.0	31.8	19.2	54.7	18.3	2.21	0.90	0.188
Metro	129	14.6	7.5	21.4	13.3	64.0	16.1	2.92	1.04	0.245
North	115	17.3	7.4	18.6	10.0	64.2	11.9	2.20	0.88	0.162
South	75	15.8	7.0	19.3	9.9	64.9	12.3	2.25	0.95	0.174
West	99	15.6	7.0	15.9	12.4	68.5	13.3	2.16	0.89	0.120
**TOTAL**	**642**	**15.2**	**7.2**	**21.2**	**14.4**	**63.7**	**15.2**	**2.4**	**0.98**	

The island average corresponds to the sum of the weighted contribution of each municipality. Weights were calculated from Martínez-Cruzado et al. (2005) [29]. SES values correspond to low (1), medium low (2), medium (3), medium high (4), and high (5). Regions are defined in Figure S1.

In addition to variation between individuals, we observed geographical differences in ancestry. Mean admixture proportions showed significant differences both at regional and municipal levels (p<10^−4^ for European and African, p<0.01 for Native American, Table 1 and Figure 1C). These geographic differences were driven by the variation in African ancestry: 23.9% of variance in African ancestry was between regions versus within regions, compared to 14.6 and 3.1% for European and Native American contributions, respectively (ANOVA p<0.001 for all comparisons). We noted a strong negative correlation between African and European ancestries (r = −0.89), but weak correlations between Native American ancestry and either European ancestry or African ancestry (−0.33 and −0.14, respectively). The proportion of African ancestry in the eastern part of the island (31.8%) is substantially higher than the island average (21.2%, p<10^−4^). Four out of the five municipalities with the highest African contribution are located in this part of the island (Figure 1C). After excluding the Eastern region, the proportion of variance between regions was reduced to 3.3%, 2.7% and 1.8% for African, European and Native American ancestries (ANOVA p<0.01 for African and European, and p = 0.06 for Native American). Admixture interpolation plots revealed the geographical patterns of variation from each individual's ancestral proportions and his or her census block (Figure 1D). An increasing west to east gradient of African ancestry is evident, with a higher African core in the area of Loíza, the municipality with the highest proportion of African ancestry (47.8%).

We confirmed the enrichment for specific ancestral components in given geographic regions of the island through spatial autocorrelation. This suite of methods tests the independence of admixture estimates from neighboring individuals. The distribution of all three ancestral components was significantly clustered (Moran's I, p<10^−4^ for all ancestries, Table S5). We further identified a clear pattern of clustering for high values of African ancestry (Getis-Ord's G, p<10^−4^, Table S5), but not for European or Native American admixture values. These results are consistent with the results of our ANOVA analysis and the ancestry distributions observed in Table 1.

The observed patterns of variation in ancestry were modeled using multiple linear regression; 14.4% of the variance in African ancestry across the island could be explained by longitude, latitude and elevation. This percentage was reduced to 8.9% and 4.8% (adjusted R^2^) when the analyses were stratified for the East region and the rest of the island, respectively.

### Historical factors influencing the geographic distribution of admixture

We used linear regression to assess the relationship between genetic ancestry and several geographical predictor variables associated with historic elements. We first tested the hypothesis that African ancestry was influenced by historical geographic factors associated with the African slave trade and the colonial sugar-based economy. Simple linear regression models demonstrated that all variables linked to geographic features of the colonial sugar plantation system were significantly associated with the distribution of African ancestry (see Text S1 and Table S6). However, the location of ports used to import African slaves to Puerto Rico did not play a significant role and were excluded from further models.

**Figure 2:**
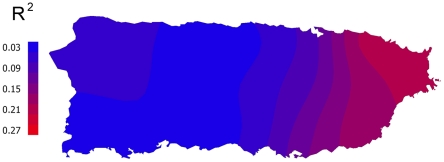
Goodness of fit of the regression model of historic variables on African ancestry. Local R^2^ values were calculated using a geographically weighted regression (GWR) model [48] and interpolation plots showed geographical variation in the accuracy of the regression model.

We built multiple linear regression models using stepwise backwards deletion and including main effects only (no interaction terms). In the final model, African ancestry was higher when individuals lived closer to historical sugar mills or in areas with a low historical production of molasses (Table 2). In addition, distance to the coast was inversely associated with African ancestry, suggesting a lack of migration of African slaves or their descendents inland. Keeping other variables fixed, individuals living 10 km inland had 14% less African ancestry than people living in the coast. This model was highly significant (p<10^−4^) and explained 7.5% of the variation in African ancestry levels between individuals (adjusted R^2^). However, the percentage of variance explained by these variables was considerably higher in the Eastern part of the island than in the rest, as evidenced through a geographically-weighted regression (Figure 2).

**Table 2:** Variation in African ancestry modeled by historical and geographical variables using multiple linear regression.

All island				
Adjusted R^2^ = 0.075				
	β estimate	S.E.	t	P
(Intercept)	−0.5676	0.02727	−20.81	<10^−4^
Distance to sugar mills (km)	−0.00195	0.00075	−2.59	0.0097
Distance to coast (km)	−0.00646	0.00145	−4.46	<10^−4^
Molasses production (m^3^/km)	−0.00141	4.0 · 10^−5^	−4.42	<10^−4^

East				
Adjusted R^2^ = 0.187				
	β estimate	S.E.	t	P
(Intercept)	−0.0556	0.10678	−0.52	0.60
Distance to sugar mills (km)	−0.0280	0.00522	−5.36	<10^−4^
Sugar production (ton/km)	−0.0032	0.00101	−3.13	0.002
Molasses production (m^3^/km)	0.0191	0.00637	2.99	0.003

No East				
Adjusted R^2^ = 0.025				
	β estimate	S.E.	t	P
(Intercept)	−0.7128	0.02592	−27.50	<10^−4^
Molasses production (m^3^/km)	−0.00107	0.00031	−3.39	0.001
Elevation (m)	−0.00015	6.4 · 10^−5^	−2.30	0.022

African ancestry is log10-transformed to assume a linear fit and satisfy model assumptions (e.g. normality of errors). Separate models for the whole island, the East region and the other 5 regions combined have been constructed. For a detailed description of the models and the variables, see Text S1 and Tables S3.

Thus, we built separate models for the East region and the rest of the island that explained 18.7% and 2.5% of the variance in the levels of African ancestry, respectively (Table 2). As observed for the whole island, African ancestry was higher among individuals in the East that lived closer to historical sugar mills and this variable had an effect an order of magnitude higher than in the whole island. Areas with a low historical production of sugar or with a high historical production of molasses presented also higher levels of African ancestry. Conversely, when individuals from the East were excluded from the analyses, elevation from sea level and historical production of molasses had a small negative impact on African ancestry. Results for all these models remained robust after 10,000 bootstrap iterations.

Finally, while historical accounts suggested that the mountainous Central Range of the island was a shelter for the Taínos, we found no evidence of associations between geographic variables such as elevation from sea level or distance to the coast and Native American ancestry.

### Socioeconomic status (SES) distribution in Puerto Rico

**Figure 3:**
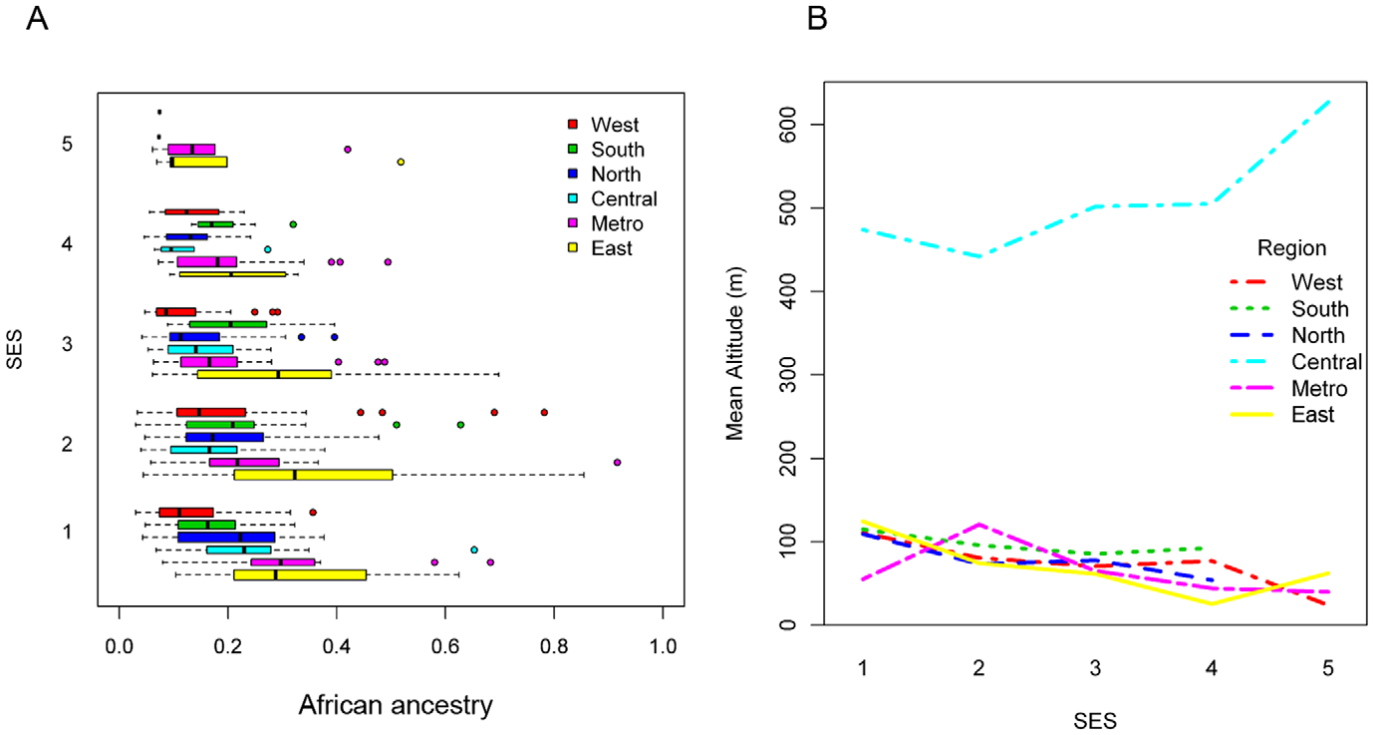
Distribution of socioeconomic status (SES) across regions. (A) Boxplots comparing African ancestry between regions by SES category. (B) Mean elevation (in meters) by geographical region and SES category.

We classified the individuals into 5 SES categories using demographic information collected at each household (see Text S1). The average SES for the entire sample of Puerto Rico was between medium and medium-low (2.4±1.0, Table 1). There was heterogeneity in the distribution of SES values across the island with significant differences both at the regional and municipality levels (p<10^−4^, Figure 3A). The metropolitan region of San Juan showed higher SES and greater variance compared to other regions. Only two municipalities presented an average SES of medium status or higher (SES ≥3), both in the Metropolitan region around San Juan (Table S4). Spatial autocorrelation analysis demonstrated that the distribution of SES was geographically clustered (Table S5).

SES was correlated with both African and European ancestries (p<10^−4^), but not with Native American ancestry. The correlation was positive for the European component (r = 0.16, indicating that higher European ancestry correlated with higher SES) and negative for African (r = −0.17). We also observed a positive association between SES and longitude, indicating increasing values of SES from West to East (p = 0.015). This is in stark contrast to the ancestry gradient, where African ancestry also increases from West to East. The overall negative correlation between African ancestry and SES is attenuated compared to the correlation at a local level because of the inverse geographic gradients. Four out of the six regions showed stronger correlation values than across the island (r = −0.20 to −0.35).

We used multiple ordinal logistic regression analyses to predict categories of SES and identify explanatory genetic and geographic variables. Our final model was highly significant (p<10^−4^) and explained a substantial proportion of variation in SES among individuals (pseudo R^2^ = 0.13). Significant variables in the final model included African ancestry, elevation and geographic region (Table 3 and Text S1). African ancestry was inversely associated with SES, with every 10% increase in African ancestry having an odds ratio (OR) of 1.28 for being in a lower SES category (95% CI 1.15-1.43; p<10^−4^). Similarly, there was also an inverse association between elevation and SES (Figure 3B), with an OR for being in a lower SES category of 1.33 for every increase of 100 meters in elevation (95% CI, 1.14-1.54; p = 0.0002). Individuals in the Central and Metropolitan regions had significantly higher SES than in the Northern region, with nearly a four-fold increased likelihood of being in a higher SES category. These higher SES values of the Central and Metropolitan regions held in comparisons with any other region. Results of the model held after 10,000 bootstrap resamples.

**Table 3:** Ordinal logistic regression model to explain the variation in SES levels from genetic ancestry and geographical variables.

		OR	95% CI	Wald Z	P
(Intercept) y> = 2				8.53	<10^−4^
(Intercept) y> = 3				−0.59	0.5545
(Intercept) y> = 4				−7.45	<10^−4^
African ancestry ¶		0.78	0.70/0.87	−4.51	<10^−4^
Elevation †		0.75	0.65/0.88	−3.66	0.0002
Region §	Central	4.10	1.87/9.01	3.52	0.0004
	East	1.39	0.86/2.26	1.35	0.1776
	Metro	4.11	2.56/6.61	5.84	<10^−4^
	South	0.99	0.60/1.63	0.15	0.8833
	West	1.04	0.60/1.81	−0.04	0.9686

Odds ratios (OR) and 95% confidence intervals (95% CI) for every independent variable are calculated for an increase in one SES category. For a detailed description of the model, see Text S1.

¶ OR is for every 10% increase in African ancestry.

† OR is for every increase of 100 meters in elevation.

§ North is the baseline region

## Discussion

Our work demonstrates that genetic admixture has substantial geographic heterogeneity even within a small geographic region like Puerto Rico. We found that the geographic patterns of African, European, and Native American ancestry throughout Puerto Rico can be explained by historic and social factors that have taken place during and since the recent colonial period. We similarly found geographic patterning of SES across the island that did not mimic the ancestry distributions. This complexity has important implications for understanding the genetic history, social dynamics and distribution of health and disease in this population.

With the exception of a gold rush in the first decades of colonization, the economy of Puerto Rico primarily consisted of large-scale sugar production in a process similar to most of the Caribbean islands at that time [27], [28]. This triggered the importation of African slaves and their descendents continued in the industry after the abolition of slavery in 1873. Thus, the location of sugar mills and sugar production variables explain a substantial proportion of the differences in African ancestry observed in present day Puerto Rico. These factors also result in an East to West gradient in the proportion of African ancestry that has been previously described for mtDNA information [29]. Sugarcane plantations were mostly located in coastal lowlands, which may explain why African ancestry decreases with distance from the coast.

The Spanish colony that imported Africans as forced labor also established a social structure to preserve the status quo of a European-descent ruling class. The African and African-admixed classes were kept in a subservient position, whether slave or free, and social class endogamy was enforced by formal laws that prevented “unequal” marriages [30]. Effects of this social stratification have led to a genetic and social structure, which continues to exist in current generations of Puerto Ricans. Recently, we detected assortative mating based on African/European ancestry among Puerto Ricans living in the island and in the mainland U.S. [12]. Here, we demonstrated that African ancestry is associated with lower SES, reinforcing the evidence that social perception influences not only social interactions and mating choice but also social position and class within society [20], [21]. In addition, socioeconomic status is independently influenced by geography, with differences between and within regions in a pattern that is actually similar to the African ancestry cline.

More than 130 years after the abolition of slavery and the legal guarantee of freedom of movements in 1873, elements associated with the use of an enslaved work force in a colonial economy can still explain the distribution of African ancestry in Puerto Rico. Census reports from 1899 and 1950 demonstrate patterns of African ancestry almost identical to those shown in Figure 1D [17], [31]. Some spatial continuity from the slave period could be expected in the first years after the abolition as most slaves were hired by their previous owners [27]. However, our results demonstrate that the descendents of slaves remained in the same areas where their ancestors resided 5-6 generations ago or moved to nearby locations. This clustered distribution of ancestry is remarkable given the relatively small size of the island, a maximum of 180 km by 64 km, and the regular migration flows between Puerto Rico and the mainland U.S. In the East region, the remnants of the original slave economy can still be seen and explain a substantial proportion of the geographical variation in African ancestry. However, it is also clear that admixed individuals with African ancestry also now occupy all regions of the island, reflecting migrations and intermarriages that have occurred over the same time period, with a residual cline of decreasing African ancestry from east to west.

Conversely, the contribution of the original Native American inhabitants of Puerto Rico, the Taínos, is not explained by geographical factors. Some authors have postulated that, after their emancipation as slaves in 1542, Taínos sought shelter in the mountainous parts in the center of the island and were slowly assimilated through the following centuries [32]. However, variation in Taíno contribution is neither higher in the Central region nor explained by distance to the coast or elevation as would be expected by the “mountain shelter” hypothesis.

Moreover, it is notable that Native American genetic ancestry does not correlate with social indices (e.g. SES). Socioeconomic differences between individuals are correlated with African and European ancestral contributions, but not with Native American. This ancestral component shows the smallest degree of variation between individuals (SD = 7.2%, Table 1). This can be explained by the fact that Taínos were the oldest ancestral population on the island and little to no Native American immigrants have arrived since active colonization began in 1508, in contrast to European and African ancestries. The lack of social importance of Native American ancestry among Puerto Ricans has also contributed to its small variation across the island and across individuals because mate choice was not related to degree of Taíno ancestry. Although the real level of variation could have been underestimated due to the markers used, our set of AIMs was informative to differentiate Native American ancestry from the other ancestral components (see Text S1). Moreover, other studies have published similar levels of variation in Native American ancestry among Puerto Ricans using genomewide information [14]. Previous investigations among Puerto Ricans have underscored the lack of social importance of Native American ancestry in processes such as assortative mating and the relationships between ancestry and social stress are based on perceived levels of European and African ancestry only [12], [23]. The fact that average Native American ancestry among Puerto Ricans is not much less than average African ancestry yet shows a much smaller variance among individuals reinforces the far more significant social role of African ancestry compared to Native American ancestry in this population. In contrast, in other Latin American countries Native American ancestry plays a key role in all these social processes [12], [33].

Another important observation is the sex-biased admixture in Puerto Rico. In a previous article using this same census-based sample, we reported that mtDNA lineages were 61.3% Native American, 27.2% African, and 11.5% European [29]. This distribution demonstrates an excess of ancestry contribution from European males and Native American females. This is a common feature in the ancestral gene pool of Latin American populations [9], [34]. Interestingly we did not observe a substantial bias for the African ancestry.

The geographic heterogeneity in genetic variation identified in this study has important implications for the identification of variants associated with disease or other clinically relevant outcomes. We have shown that variation in ancestry proportions can lead to bias in association studies [11]. It has been postulated that carefully matching cases and controls by geographical origin could minimize the problem of population stratification in human populations, but even modest levels of genetic structure within a population can lead to false positive and false negative results [11], [35]. Moreover, variation in SES can also confound genetic association results [33]. In Puerto Rico, SES and African ancestry increase from west to east, but they are inversely correlated irrespective of location. This is evidence that the relationship between ancestry and SES is a local phenomenon within a region and not across regions since the trends are in opposite directions. If these geographic patterns of SES and African ancestry are not considered when selecting samples, they could confound association results.

As we have shown, ancestry differences are associated with social differences and, in turn, social processes such as assortative mating discourage individuals from choosing potential mates of different ancestry. This process helps to maintain genetic stratification within Puerto Rico. The large variation in individual admixture estimates that we observe here has been previously reported for different populations across Latin America [9], [11], [36]. In addition, the observed correlations between genetic ancestry and social indices have been consistently described for populations across the American continent [19], [20], [21]. Thus, it is important to be mindful of genetic and social structures when carrying out biomedical research in Hispanic/Latino populations.

The microgeographic approach integrating different sources of information (e.g. genetic, geographic, historic, and social) could be relevant to detect founder effects that may influence disease prevalence. Among Hispanic/Latinos, some founder effects have already been identified with rare diseases such as Bloom Syndrome and Hermansky Pudlak Syndrome [37], [38], and other founder effects have been associated with an elevated incidence of highly penetrant mutations for diseases such as breast cancer [39], [40]. Furthermore, in the near future we will be able to use genomewide information to reconstruct demographic events at an unprecedented fine scale. This will enable us to identify events such as migrations, kinship relations or time of arrival of ancestors to a certain population, which could be complemented by the addition of census and historical registry data. Most studies in human genetics have focused on comparisons between groups, but a complete understanding of the historical, social interactions and disease processes will require the analysis of spatial and temporal interactions between individuals and their environment.

## Materials and Methods

### Dataset

A census-based sample of 800 individuals from Puerto Rico (see Text S1 and Martinez-Cruzado et al. [29] for sample details) was genotyped for a panel of 106 Ancestry Informative Markers (AIMs) selected for their informativeness to differentiate between the three ancestral groups: West Africans, Europeans, and Native Americans. Written informed consent was obtained from all participants and approved by local institutional review boards. Complete details on this panel of AIMs and subjects representing the ancestral populations have been previously described [41]. After quality control, data for 93 AIMs on 642 participants was included in final analyses (see Text S1 and Table S1). For every individual, we obtained socioeconomic status (SES), geographic location at the census block level, elevation from sea level, distance to the nearest coast, and distance to several historical elements related to the African slave trade and the colonial sugar economy (see Text S1 and Tables S2 and S3) [27], [42], [43].

### Admixture estimates

We combined our Puerto Rican samples in this study with data for the same AIM panel on 37 West African, 42 European, and 30 Native American individuals. Individual ancestral estimates (IAE) were calculated using the Bayesian clustering algorithms in STRUCTURE [25], [26]. We ran an admixture model for 20,000 burn-ins and 20,000 further iterations, assuming three ancestral populations (K = 3) and allowing 15 generations since the admixture event took place.

### Geographic analyses

Geostatistical methods were used to analyze spatial pattern of variation in admixture and SES. We tested the independence of admixture and SES estimates from neighboring individuals by means of spatial autocorrelation, specifically Moran's I and Getis and Ord's G statistics [44], [45] using the ArcView GIS 9.3.1 software (ESRI, Redlands, California, USA). We used MapViewer 6 software (Golden Software Inc., Golden, CO) with 50 nodes to construct contour plots of admixture across Puerto Rico based on the underlying pattern of spatial correlation using the Kriging estimation method and a linear variogram model [46]. Exponential and gaussian variogram models revealed similar patterns of spatial distribution [47]. Geographical differences in SES and ancestry were also assessed using ANOVA and Pearson's chi-square tests. Relationships between ancestry components, longitude, latitude, altitude and SES were assessed using Pearson's correlation. We applied multiple linear regression models to estimate the proportion of variation in ancestry that could be explained by geographic and historical factors. Geographically weighted regression models (GWR) were used to estimate local R^2^ values of the regression models for variation in ancestry explained by historical variables [48]. Ordinal logistic models were implemented to analyze the variation in SES that was explained by geography and genetic ancestry. All these calculations were performed using the R and Python programming languages.

## Supporting Information

Figure S1 Location in Puerto Rico of different elements used in the present study. (A) Municipalities of Puerto Rico included in this study coloured according to the different regions used for study purposes. (B) Districts holding sugar plantations during the 19th century and used to collect sugar-related variables in Table S3. The geographically small district of San Juan, which lacked sugar plantations, is merged to the district of Bayamón.
(TIF)

Table S1 Genomic position and allele frequency of the 93 AIMs used in the present study.
(DOC)

Table S2 List of the 128 census blocks sampled in the present study with information on their municipality, number of samples included in the final analyses, geographic location (in decimal long/lat coordinates) and altitude from sea level (in meters)
(DOC)

Table S3 Sugarcane plantation area and production of sugar and molasses per district in 1830. Areas covered by each district are shown in Figure S1B. Original units measured area in cuerdas (1 cuerda  = 0.393 ha), weight in quintales (1 quintal  = 46.01 kg) and volume in cuartillos (1 cuartillo  = 0.504 l).
(DOC)

Table S4 Admixture estimates and SES per region and municipality. The island average corresponds to the sum of the weighted contribution of each municipality. Weights were calculated from Martínez-Cruzado et al. (2005).
(DOC)

Table S5 Spatial autocorrelation results for individual ancestry estimates (IAE) and socioeconomic status (SES).
(DOC)

Table S6 Results from a simple linear regression model between African ancestry and historical and geographic variables. African ancestry is log10-transformed to satisfy model assumptions (normality of errors, …). For a detailed description of the variables, see Text S1 and Table S3.
(DOC)

Text S1 (DOC)
