# Supplementary figures and images for: History Shaped the Geographic Distribution of Genomic Admixture on the Island of Puerto Rico
Source: PLoS One. 2011 Jan 31;6(1):e16513. doi: 10.1371/journal.pone.0016513 (PMC3031579; doi:10.1371/journal.pone.0016513)

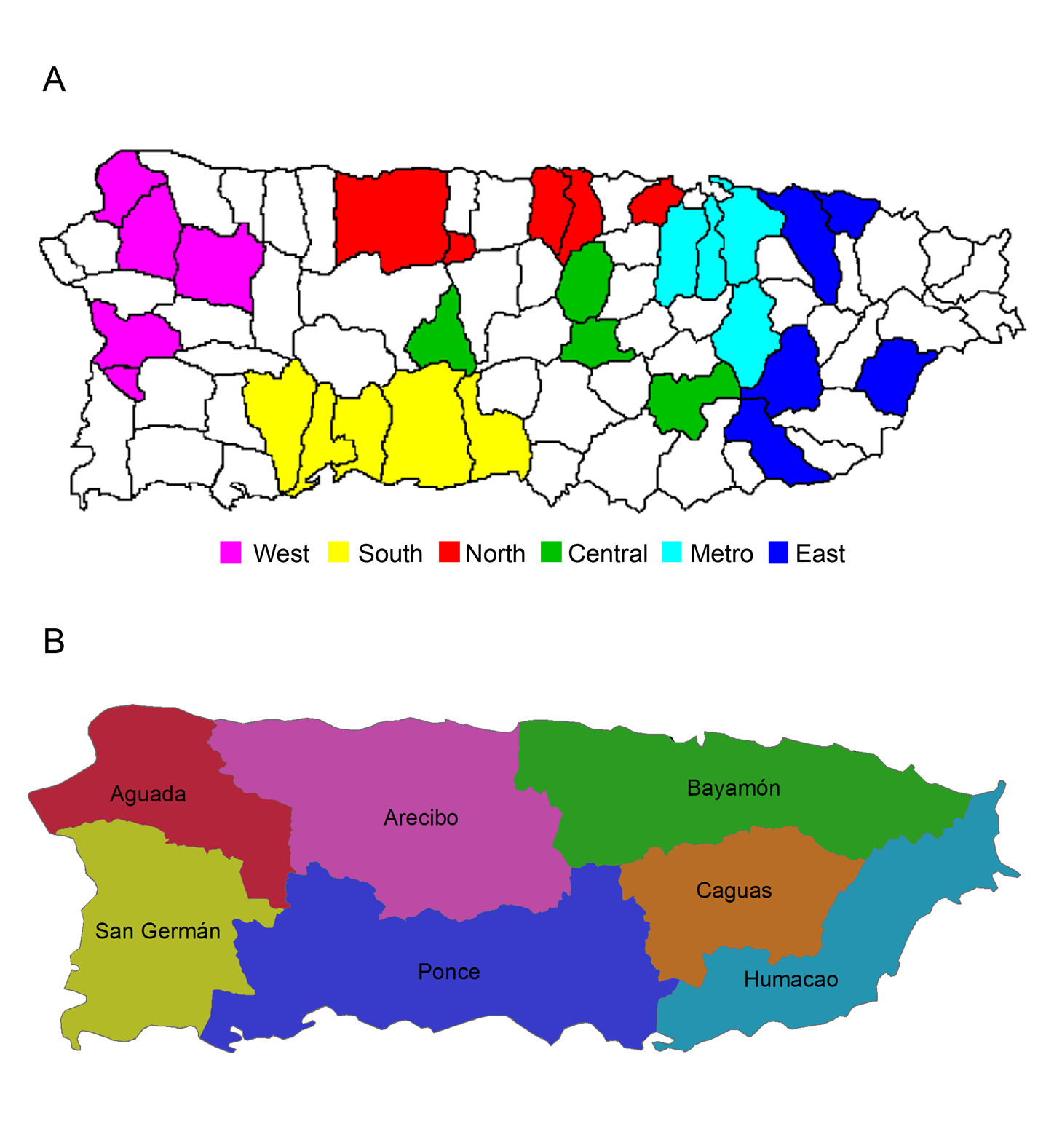

Supplement: Figure S1 — Location in Puerto Rico of different elements used in the present study. (A) Municipalities of Puerto Rico included in this study coloured according to the different regions used for study purposes. (B) Districts holding sugar plantations during the 19th century and used to collect sugar-related variables in Table S3. The geographically small district of San Juan, which lacked sugar plantations, is merged to the district of Bayamón. (TIF) [file pone.0016513.s001.tif]
