# Supplementary material for: History Shaped the Geographic Distribution of Genomic Admixture on the Island of Puerto Rico
Source: PLoS One. 2011 Jan 31;6(1):e16513. doi: 10.1371/journal.pone.0016513 (PMC3031579; doi:10.1371/journal.pone.0016513)
Supplement: Table S1 — Genomic position and allele frequency of the 93 AIMs used in the present study. (DOC) [file pone.0016513.s002.doc]

Table S1. Genomic position and allele frequency of the 93 AIMs used in the present study.

|  |  |  |  |  |  | **Allele 1 Freq** | | |
| --- | --- | --- | --- | --- | --- | --- | --- | --- |
| **rs number** | **Chr** | **Phys. Location** | **Cytoband** | **Allele 1** | **Allele 2** | **African** | **European** | **Native American** |
| rs1004704 | 16 | 48315382 | 16q12.1 | A | G | 0.028 | 0.214 | 0.867 |
| rs10131076 | 14 | 78764426 | 14q31.1 | A | G | 0.694 | 0.071 | 0.000 |
| rs1013459 | 18 | 11690534 | 18p11.21 | A | G | 0.233 | 0.900 | 0.967 |
| rs10214949 | 7 | 78660644 | 7q21.11 | A | G | 0.281 | 0.845 | 0.967 |
| rs10248051 | 7 | 50861102 | 7p12.1 | C | T | 0.083 | 0.833 | 0.200 |
| rs1036543 | 2 | 133886983 | 2q21.2 | C | T | 0.750 | 0.024 | 0.767 |
| rs10484578 | 6 | 35293174 | 6p21.31 | A | G | 0.944 | 0.375 | 0.067 |
| rs10486576 | 7 | 27861415 | 7p15.1 | A | G | 0.944 | 0.900 | 0.200 |
| rs10488172 | 7 | 132751390 | 7q33 | A | C | 0.972 | 0.786 | 0.200 |
| rs10491097 | 17 | 19523240 | 17p11.2 | C | T | 0.056 | 0.647 | 0.133 |
| rs10491654 | 9 | 97519365 | 9q22.33 | A | G | 0.500 | 0.286 | 0.900 |
| rs10492585 | 13 | 103084177 | 13q33.2 | A | G | 0.100 | 0.940 | 1.000 |
| rs10497705 | 2 | 190694557 | 2q32.2 | C | T | 0.118 | 0.238 | 0.867 |
| rs10498255 | 2 | 231814769 | 2q37.1 | A | G | 0.250 | 0.810 | 0.967 |
| rs10500505 | 16 | 64718164 | 16q21 | A | T | 0.056 | 0.214 | 0.800 |
| rs10501474 | 11 | 80126955 | 11q14.1 | C | T | 0.056 | 0.643 | 0.800 |
| rs10506816 | 12 | 78427325 | 12q21.2 | A | T | 0.861 | 0.000 | 0.100 |
| rs10508349 | 10 | 8302970 | 10p14 | A | G | 0.056 | 0.012 | 0.767 |
| rs10510791 | 3 | 57251433 | 3p14.3 | C | G | 0.972 | 0.488 | 0.133 |
| rs10515535 | 5 | 143544652 | 5q32 | A | G | 1.000 | 0.286 | 0.133 |
| rs10515919 | 2 | 75515133 | 2p12 | A | G | 0.861 | 0.881 | 0.167 |
| rs10517518 | 4 | 61798796 | 4q13.1 | C | T | 0.944 | 0.857 | 0.133 |
| rs10519979 | 4 | 150212578 | 4q31.23 | A | G | 0.167 | 0.451 | 0.967 |
| rs10520440 | 4 | 181494895 | 4q34.3 | G | T | 1.000 | 0.786 | 0.167 |
| rs10520678 | 15 | 86667051 | 15q25.3 | C | T | 0.154 | 0.732 | 1.000 |
| rs1073319 | 2 | 29414989 | 2p23.2 | C | T | 0.917 | 0.810 | 0.167 |
| rs12953952 | 18 | 65886896 | 18q22.2 | C | T | 0.139 | 0.927 | 0.967 |
| rs1353251 | 5 | 35902708 | 5p13.2 | C | T | 0.972 | 0.798 | 0.267 |
| rs138022 | 22 | 38856075 | 22q13.1 | A | G | 0.833 | 0.262 | 0.033 |
| rs1397618 | 10 | 120497262 | 10q26.11 | A | T | 0.294 | 0.952 | 1.000 |
| rs1398829 | 4 | 21774158 | 4p15.31 | A | T | 0.222 | 0.976 | 1.000 |
| rs1451928 | 14 | 46330779 | 14q21.3 | A | C | 0.861 | 0.857 | 0.200 |
| rs1470524 | 2 | 45104050 | 2p21 | C | T | 0.222 | 0.786 | 0.533 |
| rs1477277 | 5 | 180784684 | 5q35.3 | C | G | 1.000 | 0.345 | 0.300 |
| rs1498991 | 3 | 20875097 | 3p24.3 | C | G | 0.944 | 0.976 | 0.250 |
| rs1517634 | 2 | 224386024 | 2q36.1 | A | G | 0.833 | 0.833 | 0.000 |
| rs153898 | 5 | 94262695 | 5q15 | A | G | 0.083 | 0.738 | 0.033 |
| rs1898280 | 8 | 116031043 | 8q23.3 | C | T | 0.889 | 0.238 | 0.833 |
| rs1919550 | 3 | 122685074 | 3q13.33 | A | T | 0.028 | 0.024 | 0.833 |
| rs1934393 | 1 | 48578535 | 1p33 | C | G | 0.222 | 0.842 | 0.300 |
| rs1984473 | 3 | 157132197 | 3q25.31 | C | T | 0.056 | 0.631 | 0.967 |
| rs1990745 | 5 | 103458138 | 5q21.2 | C | T | 1.000 | 0.881 | 0.233 |
| rs2042762 | 18 | 33529609 | 18q12.2 | C | T | 0.000 | 0.000 | 0.733 |
| rs2253624 | 17 | 70329204 | 17q24.3 | G | T | 0.176 | 1.000 | 1.000 |
| rs2296274 | 14 | 59907219 | 14q23.1 | A | G | 0.118 | 0.750 | 0.967 |
| rs249847 | 12 | 97370184 | 12q23.1 | C | T | 0.056 | 0.463 | 0.967 |
| rs257748 | 5 | 15872353 | 5p15.1 | A | T | 0.806 | 0.381 | 0.967 |
| rs2592888 | 1 | 156802365 | 1q23.2 | A | G | 0.088 | 0.762 | 1.000 |
| rs2595456 | 11 | 6849072 | 11p15.4 | A | G | 0.306 | 0.524 | 0.000 |
| rs2711070 | 2 | 159705078 | 2q24.1 | C | G | 0.139 | 0.464 | 0.967 |
| rs2785279 | 10 | 33713882 | 10p11.22 | C | T | 0.824 | 0.262 | 0.067 |
| rs2817611 | 1 | 11322709 | 1p36.22 | C | T | 0.278 | 0.952 | 0.967 |
| rs2829454 | 21 | 25194942 | 21q21.2 | C | T | 0.056 | 0.274 | 0.933 |
| rs2840290 | 9 | 16723957 | 9p22.2 | C | T | 0.861 | 0.268 | 0.900 |
| rs304051 | 3 | 4553306 | 3p26.1 | C | T | 0.750 | 0.548 | 0.000 |
| rs354747 | 20 | 59598070 | 20q13.33 | A | G | 0.083 | 0.643 | 0.767 |
| rs3806218 | 1 | 144518860 | 1q21.1 | C | T | 0.059 | 0.667 | 0.821 |
| rs3828121 | 1 | 81844793 | 1p31.1 | A | G | 1.000 | 0.869 | 0.300 |
| rs3860446 | 2 | 104110751 | 2q12.1 | A | G | 0.972 | 0.357 | 1.000 |
| rs4013967 | 9 | 72354189 | 9q21.13 | C | T | 0.059 | 0.655 | 1.000 |
| rs4034627 | 12 | 126750571 | 12q24.32 | A | G | 0.781 | 0.048 | 0.033 |
| rs4076700 | 12 | 115795273 | 12q24.22 | A | G | 0.206 | 0.833 | 0.900 |
| rs4130405 | 8 | 99377358 | 8q22.2 | G | T | 0.000 | 0.190 | 0.800 |
| rs4130513 | 16 | 78238277 | 16q23.1 | A | G | 0.722 | 0.083 | 0.192 |
| rs4625554 | 12 | 4286565 | 12p13.32 | C | T | 0.167 | 0.298 | 0.867 |
| rs4657449 | 1 | 162652658 | 1q23.3 | A | G | 0.083 | 0.071 | 0.733 |
| rs4762106 | 12 | 64304740 | 12q14.3 | C | T | 0.806 | 0.095 | 0.667 |
| rs4852696 | 2 | 83126181 | 2p12 | C | G | 0.250 | 0.905 | 0.700 |
| rs4934436 | 10 | 90447897 | 10q23.31 | A | G | 0.694 | 0.452 | 0.967 |
| rs5000507 | 13 | 79886955 | 13q31.1 | A | T | 0.139 | 0.713 | 1.000 |
| rs567992 | 11 | 105800114 | 11q22.3 | C | T | 1.000 | 0.845 | 0.333 |
| rs6569792 | 6 | 132675321 | 6q23.2 | A | G | 0.111 | 0.679 | 0.800 |
| rs6684063 | 1 | 30201812 | 1p35.2 | A | C | 0.222 | 0.833 | 0.167 |
| rs6804094 | 3 | 188378883 | 3q27.3 | A | T | 0.972 | 0.654 | 0.133 |
| rs6883095 | 5 | 79975120 | 5q14.1 | C | T | 0.861 | 0.548 | 0.033 |
| rs6911727 | 6 | 9061397 | 6p24.3 | A | G | 0.139 | 0.476 | 1.000 |
| rs708915 | 20 | 8395667 | 20p12.3 | A | T | 0.735 | 0.100 | 0.733 |
| rs7463344 | 8 | 33921195 | 8p12 | C | G | 0.639 | 0.000 | 0.000 |
| rs7535375 | 1 | 232954308 | 1q42.3 | C | T | 0.111 | 0.714 | 0.800 |
| rs798887 | 19 | 59485000 | 19q13.42 | A | G | 0.778 | 0.854 | 0.133 |
| rs802524 | 7 | 145343383 | 7q35 | A | G | 0.278 | 0.929 | 0.967 |
| rs842634 | 2 | 61065756 | 2p16.1 | C | T | 0.972 | 0.738 | 0.233 |
| rs868179 | 2 | 177752041 | 2q31.1 | A | G | 0.700 | 0.073 | 0.000 |
| rs879780 | 11 | 129545756 | 11q24.3 | C | T | 0.667 | 0.048 | 0.000 |
| rs888861 | 19 | 40073692 | 19q13.12 | A | G | 0.000 | 0.738 | 0.900 |
| rs9292118 | 5 | 55916081 | 5q11.2 | C | T | 0.176 | 0.833 | 0.967 |
| rs9302185 | 15 | 52670920 | 15q21.3 | C | T | 0.875 | 0.190 | 0.033 |
| rs9307613 | 4 | 130816225 | 4q28.2 | A | T | 0.094 | 0.775 | 0.833 |
| rs9310888 | 3 | 29261727 | 3p24.1 | C | T | 0.667 | 0.075 | 0.000 |
| rs9320808 | 6 | 121635172 | 6q22.31 | A | G | 0.861 | 0.095 | 0.967 |
| rs9323178 | 14 | 21103774 | 14q11.2 | A | G | 0.176 | 0.452 | 0.967 |
| rs9325872 | 8 | 20490544 | 8p21.3 | A | G | 0.944 | 0.321 | 1.000 |
| rs948360 | 11 | 65882085 | 11q13.2 | C | T | 0.250 | 0.974 | 1.000 |
