# Supplementary material for: History Shaped the Geographic Distribution of Genomic Admixture on the Island of Puerto Rico
Source: PLoS One. 2011 Jan 31;6(1):e16513. doi: 10.1371/journal.pone.0016513 (PMC3031579; doi:10.1371/journal.pone.0016513)
Supplement: Table S2 — List of the 128 census blocks sampled in the present study with information on their municipality, number of samples included in the final analyses, geographic location (in decimal long/lat coordinates) and altitude from sea level (in meters) (DOC) [file pone.0016513.s003.doc]

Table S2. List of the 128 census blocks sampled in the present study with information on their municipality, number of samples included in the final analyses, geographic location (in decimal long/lat coordinates) and altitude from sea level (in meters)

| **Census Block** | **Municipality** | **N** | **Longitude** | **Latitude** | **Elevation** |
| --- | --- | --- | --- | --- | --- |
| 72005401298114 | Aguadilla | 6 | -67.1347 | 18.40336 | 55 |
| 72005401300131 | Aguadilla | 8 | -67.1254 | 18.45284 | 151 |
| 72005401300215 | Aguadilla | 5 | -67.1400 | 18.44524 | 164 |
| 72013300300719 | Arecibo | 10 | -66.6848 | 18.42587 | 22 |
| 72013301200409 | Arecibo | 4 | -66.7430 | 18.45419 | 44 |
| 72013301600182 | Arecibo | 1 | -66.7207 | 18.42276 | 131 |
| 72013302100406 | Arecibo | 2 | -66.6686 | 18.41043 | 188 |
| 72013302200207 | Arecibo | 7 | -66.6118 | 18.42797 | 116 |
| 72019952298137 | Barranquitas | 12 | -66.2883 | 18.20104 | 627 |
| 72019952298209 | Barranquitas | 9 | -66.3044 | 18.23229 | 662 |
| 72019952298223B | Barranquitas | 7 | -66.3013 | 18.19918 | 675 |
| 72021030102203 | Bayamón | 6 | -66.1294 | 18.39319 | 19 |
| 72021030904705 | Bayamón | 2 | -66.1761 | 18.39046 | 22 |
| 72021031031123 | Bayamón | 5 | -66.1618 | 18.41679 | 9 |
| 72021031101705 | Bayamón | 3 | -66.1876 | 18.36597 | 51 |
| 72021031301110 | Bayamón | 1 | -66.1883 | 18.33368 | 149 |
| 72021031302210 | Bayamón | 5 | -66.1776 | 18.36410 | 50 |
| 72021031611507 | Bayamón | 2 | -66.1505 | 18.37858 | 24 |
| 72021031701302 | Bayamón | 5 | -66.1435 | 18.36582 | 39 |
| 72021032200110 | Bayamón | 7 | -66.1842 | 18.30302 | 181 |
| 72025200800103 | Caguas | 3 | -66.0161 | 18.23121 | 59 |
| 72025202300201 | Caguas | 4 | -66.0572 | 18.22416 | 100 |
| 72025202401107 | Caguas | 5 | -66.0871 | 18.21762 | 373 |
| 72025202402413 | Caguas | 6 | -66.0742 | 18.20593 | 244 |
| 72025202500122 | Caguas | 3 | -66.0541 | 18.20610 | 110 |
| 72025202600113 | Caguas | 5 | -66.0111 | 18.21128 | 167 |
| 72031050211803 | Carolina | 2 | -65.9783 | 18.42982 | 5 |
| 72031050232114 | Carolina | 3 | -65.9797 | 18.40036 | 19 |
| 72031050242302 | Carolina | 4 | -65.9701 | 18.40223 | 7 |
| 72031050600206 | Carolina | 5 | -65.9607 | 18.38599 | 10 |
| 72031050811205A | Carolina | 7 | -65.9600 | 18.42380 | 2 |
| 72031050901124 | Carolina | 2 | -65.9676 | 18.34396 | 131 |
| 72035260198120B | Cayey | 2 | -66.0785 | 18.14442 | 527 |
| 72035260198309 | Cayey | 6 | -66.1040 | 18.12466 | 441 |
| 72035260800103 | Cayey | 10 | -66.1412 | 18.12192 | 383 |
| 72047530500107 | Corozal | 6 | -66.3075 | 18.32698 | 146 |
| 72047530500217 | Corozal | 9 | -66.3417 | 18.30355 | 305 |
| 72047530500303 | Corozal | 7 | -66.3461 | 18.32209 | 227 |
| 72054580200106 | Florida | 7 | -66.5561 | 18.35831 | 203 |
| 72054580200213 | Florida | 2 | -66.5637 | 18.35850 | 207 |
| 72054580200215 | Florida | 4 | -66.5611 | 18.36005 | 199 |
| 72054580200301 | Florida | 8 | -66.5584 | 18.34887 | 205 |
| 72059740100408C | Guayanilla | 9 | -66.8148 | 18.04251 | 109 |
| 72059740100418 | Guayanilla | 6 | -66.8003 | 18.04132 | 44 |
| 72061040302621 | Guaynabo | 1 | -66.1082 | 18.40549 | 29 |
| 72061040421206 | Guaynabo | 3 | -66.1157 | 18.37350 | 61 |
| 72061040432906 | Guaynabo | 3 | -66.1058 | 18.37336 | 39 |
| 72061040602308 | Guaynabo | 2 | -66.1223 | 18.36814 | 28 |
| 72067820100101C | Hormigueros | 13 | -67.1017 | 18.13831 | 77 |
| 72067820100121 | Hormigueros | 4 | -67.1034 | 18.13263 | 41 |
| 72067820100201A | Hormigueros | 6 | -67.1178 | 18.14671 | 62 |
| 72069180400101D | Humacao | 20 | -65.8226 | 18.16521 | 87 |
| 72069180900120A | Humacao | 5 | -65.8035 | 18.10852 | 7 |
| 72069180900204 | Humacao | 8 | -65.8186 | 18.12714 | 29 |
| 72069180900306 | Humacao | 2 | -65.8112 | 18.09465 | 23 |
| 72069180900314 | Humacao | 7 | -65.8089 | 18.08243 | 48 |
| 72073956200409 | Jayuya | 6 | -66.6326 | 18.20711 | 505 |
| 72073956200422 | Jayuya | 4 | -66.6224 | 18.19038 | 498 |
| 72073956200423 | Jayuya | 9 | -66.6248 | 18.18116 | 584 |
| 72075710198114 | Juana Díaz | 8 | -66.4418 | 18.04682 | 250 |
| 72075710901317 | Juana Díaz | 8 | -66.5355 | 18.02344 | 30 |
| 72087110300204 | Loíza | 4 | -65.8717 | 18.43445 | 4 |
| 72087110300327 | Loíza | 6 | -65.8928 | 18.42099 | 4 |
| 72087110300401 | Loíza | 7 | -65.8581 | 18.42224 | 4 |
| 72087110300403 | Loíza | 12 | -65.8671 | 18.42731 | 4 |
| 72097080500106 | Mayagüez | 2 | -67.1449 | 18.20031 | 12 |
| 72097080900703 | Mayagüez | 2 | -67.1355 | 18.19907 | 20 |
| 72097081501604 | Mayagüez | 6 | -67.1363 | 18.24299 | 22 |
| 72097081522102B | Mayagüez | 2 | -67.1524 | 18.23716 | 24 |
| 72097082022203 | Mayagüez | 5 | -67.1266 | 18.17909 | 98 |
| 72097082102101 | Mayagüez | 3 | -67.1537 | 18.17841 | 7 |
| 72099420500120 | Moca | 8 | -67.0956 | 18.36541 | 83 |
| 72099420500201 | Moca | 5 | -67.1255 | 18.37696 | 41 |
| 72099420500336 | Moca | 4 | -67.1216 | 18.32976 | 144 |
| 72109290100127 | Patillas | 5 | -66.0559 | 18.06949 | 184 |
| 72109290100130B | Patillas | 3 | -66.0701 | 18.05533 | 399 |
| 72109290100142 | Patillas | 7 | -66.0540 | 18.05109 | 158 |
| 72109290100303B | Patillas | 3 | -66.0372 | 18.01086 | 142 |
| 72111730400117 | Peñuelas | 5 | -66.7273 | 18.05575 | 66 |
| 72111730400310 | Peñuelas | 1 | -66.7225 | 18.05452 | 55 |
| 72113070602111 | Ponce | 1 | -66.6103 | 18.01330 | 24 |
| 72113070900104 | Ponce | 1 | -66.6244 | 18.01564 | 46 |
| 72113071500503 | Ponce | 4 | -66.6282 | 18.00084 | 8 |
| 72113071800103 | Ponce | 4 | -66.6078 | 18.01009 | 18 |
| 72113072000114 | Ponce | 1 | -66.6005 | 18.00514 | 16 |
| 72113072702430 | Ponce | 6 | -66.6168 | 18.06239 | 195 |
| 72113073001133B | Ponce | 5 | -66.6715 | 18.04897 | 96 |
| 72113073005216 | Ponce | 2 | -66.6550 | 18.01825 | 30 |
| 72127001500302 | San Juan | 1 | -66.0614 | 18.45103 | 7 |
| 72127001601206 | San Juan | 1 | -66.0640 | 18.44683 | 29 |
| 72127004300202 | San Juan | 8 | -66.0690 | 18.42964 | 0 |
| 72127005000402 | San Juan | 6 | -66.0259 | 18.40873 | 15 |
| 72127005103102 | San Juan | 3 | -66.0248 | 18.40295 | 21 |
| 72127005401206A | San Juan | 6 | -66.0038 | 18.38560 | 58 |
| 72127005403804B | San Juan | 3 | -66.0162 | 18.38994 | 41 |
| 72127006700201C | San Juan | 4 | -66.0580 | 18.41112 | 20 |
| 72127006800412 | San Juan | 1 | -66.0682 | 18.41397 | 10 |
| 72127007300701 | San Juan | 4 | -66.0956 | 18.41248 | 29 |
| 72127007800505 | San Juan | 4 | -66.0950 | 18.39494 | 30 |
| 72127008100101 | San Juan | 2 | -66.0937 | 18.38748 | 40 |
| 72127009111101A | San Juan | 3 | -66.0343 | 18.39655 | 37 |
| 72127009112201A | San Juan | 5 | -66.0352 | 18.38598 | 64 |
| 72127009300304 | San Juan | 2 | -66.0481 | 18.39334 | 71 |
| 72127009800102 | San Juan | 3 | -66.0738 | 18.38057 | 40 |
| 72127009902408 | San Juan | 1 | -66.0844 | 18.35193 | 84 |
| 72127010012401 | San Juan | 1 | -66.0419 | 18.37143 | 43 |
| 72129220400139 | San Lorenzo | 5 | -65.9558 | 18.18406 | 101 |
| 72129220400152 | San Lorenzo | 6 | -65.9496 | 18.19186 | 104 |
| 72129220400203 | San Lorenzo | 6 | -65.9575 | 18.17976 | 90 |
| 72129220400239 | San Lorenzo | 8 | -65.9272 | 18.13365 | 333 |
| 72131958700249 | San Sebastián | 6 | -67.0343 | 18.34625 | 48 |
| 72131958700307 | San Sebastián | 6 | -67.0176 | 18.35452 | 74 |
| 72131959200341 | San Sebastián | 8 | -66.9641 | 18.28032 | 188 |
| 72137120300104 | Toa Baja | 2 | -66.1809 | 18.45327 | 1 |
| 72137120400202 | Toa Baja | 1 | -66.1835 | 18.45823 | -1 |
| 72137120900102 | Toa Baja | 3 | -66.1712 | 18.43589 | 4 |
| 72137121000101 | Toa Baja | 8 | -66.1864 | 18.44569 | 1 |
| 72137122100101 | Toa Baja | 2 | -66.2480 | 18.40436 | 93 |
| 72143550200103 | Vega Alta | 8 | -66.3283 | 18.44354 | 45 |
| 72143550200107C | Vega Alta | 12 | -66.3293 | 18.42897 | 71 |
| 72143550200201 | Vega Alta | 3 | -66.3453 | 18.44938 | 7 |
| 72143550200301 | Vega Alta | 6 | -66.3414 | 18.44149 | 40 |
| 72145560700102 | Vega Baja | 4 | -66.3870 | 18.43663 | 63 |
| 72145560700209 | Vega Baja | 9 | -66.3960 | 18.43866 | 66 |
| 72145560800103 | Vega Baja | 7 | -66.4153 | 18.43581 | 67 |
| 72145560800105 | Vega Baja | 5 | -66.4236 | 18.43357 | 67 |
| 72153750600111A | Yauco | 11 | -66.8492 | 18.01698 | 123 |
| 72153750600210 | Yauco | 3 | -66.8671 | 18.01055 | 89 |
