# Supplementary material for: History Shaped the Geographic Distribution of Genomic Admixture on the Island of Puerto Rico
Source: PLoS One. 2011 Jan 31;6(1):e16513. doi: 10.1371/journal.pone.0016513 (PMC3031579; doi:10.1371/journal.pone.0016513)
Supplement: Table S3 — Sugarcane plantation area and production of sugar and molasses per district in 1830. Areas covered by each district are shown in Figure S1B. Original units measured area in cuerdas (1 cuerda = 0.393 ha), weight in quintales (1 quintal = 46.01 kg) and volume in cuartillos (1 cuartillo = 0.504 l). (DOC) [file pone.0016513.s004.doc]

Table S3. Sugarcane plantation area and production of sugar and molasses per district in 1830. Areas covered by each district are shown in Figure S1B. Original units measured area in *cuerdas* (1 cuerda = 0.393 ha), weight in *quintales* (1 quintal = 46.01 kg) and volume in *cuartillos* (1 cuartillo = 0.504 l).

| **Distrito** | **Arecibo** | **Aguada** | **Bayamón** | **Caguas** | **Humacao** | **Ponce** | **San Germán** | **Total** |
| --- | --- | --- | --- | --- | --- | --- | --- | --- |
| **Sugarcane (ha)** | 367.5 | 384.0 | 1097.4 | 132.5 | 615.9 | 822.6 | 983.8 | 4403.6 |
| **Sugar (metric tons)** | 701.7 | 582.7 | 2423.1 | 130.1 | 2126.7 | 3413.5 | 3621.0 | 12998.8 |
| **Molasses (m^3^)** | 323.7 | 81.1 | 92.0 | 35.4 | 321.9 | 299.3 | 975.6 | 2129.0 |
