# Supplementary material for: History Shaped the Geographic Distribution of Genomic Admixture on the Island of Puerto Rico
Source: PLoS One. 2011 Jan 31;6(1):e16513. doi: 10.1371/journal.pone.0016513 (PMC3031579; doi:10.1371/journal.pone.0016513)
Supplement: Table S4 — Admixture estimates and SES per region and municipality. The island average corresponds to the sum of the weighted contribution of each municipality. Weights were calculated from Martínez-Cruzado et al. (2005). (DOC) [file pone.0016513.s005.doc]

Table S4. Admixture estimates and SES per region and municipality. The island average corresponds to the sum of the weighted contribution of each municipality. Weights were calculated from Martínez-Cruzado et al. (2005).

|  |  |  | **Native American** | | **African** | | **European** | | **SES** | |  |
| --- | --- | --- | --- | --- | --- | --- | --- | --- | --- | --- | --- |
| **Region** | **Municipality** | **N** | **Mean** | **SD** | **Mean** | **SD** | **Mean** | **SD** | **Mean** | **SD** | **Weight** |
| Central |  | 87 | 14.3 | 6.1 | 16.9 | 9.1 | 68.8 | 10.2 | 2.31 | 0.85 | 0.111 |
|  | Barranquitas | 28 | 13.7 | 6.1 | 15.8 | 12.5 | 70.5 | 13.5 | 2.6 | 1.07 | 0.028 |
|  | Cayey | 18 | 12.1 | 5.4 | 17.6 | 6.5 | 70.4 | 8.8 | 2.1 | 0.80 | 0.025 |
|  | Corozal | 22 | 15.3 | 6.1 | 17.3 | 8.6 | 67.4 | 8.2 | 2.2 | 0.85 | 0.028 |
|  | Jayuya | 19 | 16.0 | 6.5 | 17.0 | 8.5 | 67.0 | 9.6 | 2.4 | 0.61 | 0.029 |
| East |  | 137 | 13.5 | 7.0 | 31.8 | 19.2 | 54.7 | 18.3 | 2.21 | 0.90 | 0.188 |
|  | Carolina | 23 | 13.5 | 7.9 | 30.3 | 22.2 | 56.2 | 19.3 | 2.8 | 0.90 | 0.054 |
|  | Humacao | 42 | 13.4 | 6.5 | 35.3 | 17.9 | 51.2 | 18.5 | 2.3 | 1.22 | 0.031 |
|  | Loíza | 29 | 11.6 | 5.5 | 47.8 | 18.5 | 40.6 | 17.4 | 1.9 | 0.35 | 0.036 |
|  | Patillas | 18 | 15.2 | 8.1 | 23.3 | 12.6 | 61.5 | 14.7 | 1.6 | 0.61 | 0.036 |
|  | San Lorenzo | 25 | 13.6 | 6.1 | 22.6 | 8.4 | 63.7 | 10.9 | 2.2 | 0.69 | 0.032 |
| Metro |  | 129 | 14.6 | 7.5 | 21.4 | 13.3 | 64.0 | 16.1 | 2.92 | 1.04 | 0.245 |
|  | Bayamón | 36 | 16.8 | 8.1 | 21.3 | 9.3 | 61.8 | 13.3 | 2.6 | 0.72 | 0.057 |
|  | Caguas | 26 | 16.2 | 6.3 | 21.3 | 9.6 | 62.6 | 12.2 | 2.5 | 0.81 | 0.037 |
|  | Guaynabo | 9 | 11.1 | 4.2 | 17.6 | 12.7 | 71.3 | 15.0 | 3.4 | 0.88 | 0.025 |
|  | San Juan | 58 | 13.8 | 7.9 | 22.2 | 15.8 | 64.0 | 18.4 | 3.1 | 1.17 | 0.126 |
| North |  | 115 | 17.3 | 7.4 | 18.6 | 10.0 | 64.2 | 11.9 | 2.20 | 0.88 | 0.162 |
|  | Arecibo | 24 | 17.3 | 7.3 | 16.7 | 7.0 | 66.0 | 8.8 | 2.0 | 0.66 | 0.029 |
|  | Florida | 21 | 18.7 | 6.4 | 14.1 | 7.9 | 67.3 | 8.1 | 2.0 | 0.92 | 0.034 |
|  | Toa Baja | 16 | 17.7 | 8.9 | 21.6 | 11.0 | 60.6 | 14.1 | 2.6 | 0.89 | 0.036 |
|  | Vega Alta | 29 | 16.2 | 7.3 | 22.1 | 12.0 | 61.7 | 14.0 | 1.9 | 0.90 | 0.035 |
|  | Vega Baja | 25 | 16.1 | 7.0 | 17.7 | 9.2 | 66.2 | 12.4 | 2.5 | 0.82 | 0.028 |
| South |  | 75 | 15.8 | 7.0 | 19.3 | 9.9 | 64.9 | 12.3 | 2.25 | 0.95 | 0.174 |
|  | Guayanilla | 15 | 18.6 | 8.5 | 17.0 | 5.4 | 64.4 | 10.7 | 1.3 | 0.49 | 0.018 |
|  | Juana Díaz | 16 | 12.0 | 4.8 | 23.5 | 14.1 | 64.5 | 15.9 | 1.8 | 0.54 | 0.021 |
|  | Peñuelas | 6 | 16.2 | 8.1 | 16.0 | 7.3 | 67.8 | 13.8 | 2.5 | 0.55 | 0.017 |
|  | Ponce | 24 | 15.4 | 6.2 | 19.7 | 11.1 | 64.8 | 12.7 | 2.4 | 1.10 | 0.044 |
|  | Yauco | 14 | 17.6 | 7.8 | 19.0 | 6.7 | 63.4 | 9.1 | 2.9 | 0.83 | 0.020 |
| West |  | 99 | 15.6 | 7.0 | 15.9 | 12.4 | 68.5 | 13.3 | 2.16 | 0.89 | 0.120 |
|  | Aguadilla | 19 | 13.5 | 6.3 | 22.7 | 20.7 | 63.8 | 18.5 | 2.0 | 0.67 | 0.037 |
|  | Hormigueros | 23 | 17.3 | 7.4 | 16.3 | 10.2 | 66.5 | 14.4 | 2.5 | 0.67 | 0.037 |
|  | Mayagüez | 20 | 16.7 | 8.6 | 16.0 | 9.2 | 67.3 | 13.7 | 2.7 | 1.14 | 0.031 |
|  | Moca | 17 | 14.1 | 5.7 | 11.1 | 5.4 | 74.8 | 6.5 | 1.8 | 0.88 | 0.036 |
|  | San Sebastián | 20 | 16.8 | 6.9 | 12.7 | 7.5 | 70.5 | 7.7 | 1.9 | 0.85 | 0.033 |
| **TOTAL** |  | **642** | **15.2** | **7.2** | **21.2** | **14.4** | **63.7** | **15.2** | **2.4** | **0.98** |  |

* SES values correspond to low (1), medium low (2), medium medium (3), medium high (4), and high (5).
