# Supplementary material for: History Shaped the Geographic Distribution of Genomic Admixture on the Island of Puerto Rico
Source: PLoS One. 2011 Jan 31;6(1):e16513. doi: 10.1371/journal.pone.0016513 (PMC3031579; doi:10.1371/journal.pone.0016513)
Supplement: Table S5 — Spatial autocorrelation results for individual ancestry estimates (IAE) and socioeconomic status (SES). (DOC) [file pone.0016513.s006.doc]

Table S5. Spatial autocorrelation results for individual ancestry estimates (IAE) and socioeconomic status (SES).

| **Inverse distance** | Moran’s I | **Z score** | **P** |
| --- | --- | --- | --- |
| African | 0.13 | 28.7 | < 10^-9^ |
| Native American | 0.02 | 4.1 | 4.7 · 10^-5^ |
| European | 0.08 | 17.5 | < 10^-9^ |
| SES | 0.04 | 9.8 | < 10^-9^ |
| **Inv. dist. squared** | **Moran’s I** | **Z score** | **P** |
| African | 0.25 | 18.6 | < 10^-9^ |
| Native American | 0.03 | 2.2 | 0.028 |
| European | 0.15 | 11.5 | < 10^-9^ |
| SES | 0.10 | 7.9 | < 10^-9^ |
| **Inverse distance** | **Getis-Ord’s G** | **Z score** | **P** |
| African | 0.0017 | 11.5 | < 10^-9^ |
| Native American | 0.0016 | 0.8 | 0.20 |
| European | 0.0016 | 1.9 | 0.03 |
| SES | 0.0016 | 6.1 | < 10^-9^ |
| **Inv. dist. squared** | **Getis-Ord’s G** | **Z score** | **P** |
| African | 0.0018 | 8.2 | < 10^-9^ |
| Native American | 0.0016 | 0.05 | 0.48 |
| European | 0.0016 | 1.2 | 0.11 |
| SES | 0.0016 | 2.3 | 0.011 |
