# Supplementary material for: History Shaped the Geographic Distribution of Genomic Admixture on the Island of Puerto Rico
Source: PLoS One. 2011 Jan 31;6(1):e16513. doi: 10.1371/journal.pone.0016513 (PMC3031579; doi:10.1371/journal.pone.0016513)
Supplement: Table S6 — Results from a simple linear regression model between African ancestry and historical and geographic variables. African ancestry is log10-transformed to satisfy model assumptions (normality of errors, …). For a detailed description of the variables, see Text S1 and Table S3. (DOC) [file pone.0016513.s007.doc]

Table S6. Results from a simple linear regression model between African ancestry and historical and geographic variables. African ancestry is log10-transformed to satisfy model assumptions (normality of errors, ...). For a detailed description of the variables, see SI text and Table S3.

| **Variable** | **P** |
| --- | --- |
| Distance to coast (km) | < 10^-4^ |
| Elevation from sea level (m) | 0.0001 |
| Distance to closest sugar mill (km) | 0.0002 |
| Sugar production (ton/km) | 0.031 |
| Molasses production (m^3^/km) | < 10^-4^ |
| Distance to slave ports (km) | 0.7712 |
