# Supplementary material for: History Shaped the Geographic Distribution of Genomic Admixture on the Island of Puerto Rico
Source: PLoS One. 2011 Jan 31;6(1):e16513. doi: 10.1371/journal.pone.0016513 (PMC3031579; doi:10.1371/journal.pone.0016513)
Supplement: Text S1 — (DOC) [file pone.0016513.s008.doc]

**History shaped the geographic distribution of genomic admixture on the island of Puerto Rico**

Marc Via^a,b,1^, Chistopher R Gignoux^b^, Lindsey A Roth^a^, Laura Fejerman^b,c^, Joshua Galanter^a^, Shweta Choudhry^b,d^, Gladys Toro-Labrador^e^, Jorge Viera-Vera^f^, Taras K Oleksyk^e^, Kenneth Beckman^g^, Elad Ziv^b,c^, Neil Risch^b,h^, Esteban González Burchard^a,b,i,1,2^, Juan Carlos Martínez-Cruzado^e,1,2^

**TEXT S1**

**Samples**

We analyzed a random sample of 800 individuals representative of the resident population of Puerto Rico. The sampling frame was based on the 1990 Census of Population and Housing and designed by the Center for Applied Social Research (University of Puerto Rico, Mayagüez). The sample included 28 of the 76 municipalities in the island of Puerto Rico, and these were selected to cover all regions in the island. The municipalities with the 8 biggest populations were selected with probability equal to one. To select the remaining 20 municipalities, the island was divided into 5 geographic regions. The municipalities within each region were stratified according to population size into 4 groups and then selected at random from each stratum with probability proportional to population size. Census blocks within each selected municipality were selected in a similar fashion, whereas housing units within census blocks were selected by systematic sampling with random starting points. The sampling frame has been previously described in more detail [1]. Written informed consent was obtained from all participants and approved by local institutional review boards.

**Genetic analyses**

Sample collection and DNA extraction were performed as in [2]. A panel of 106 Ancestry Informative Markers (AIMs) was genotyped in the Puerto Rican samples to estimate their individual admixture proportions from the three ancestral populations: West Africans, Europeans, and Native Americans. These AIMs were selected from the Affymetrix 100K SNP chip (Affymetrix, Santa Clara, CA) because they were informative to differentiate between ancestral groups (difference in allele frequency (δ) of at least 0.5 between any two ancestral populations), adequately distributed across the genome, and not in linkage disequilibrium among them. Complete details on this panel of AIMs have been previously described [3]. Previous works have demonstrated that approximately 100 AIMs provide estimates of individual ancestry that closely correlate (> 0.9) the true ancestral proportions [4].

Genotyping of AIMs was done using iPLEX reagents and protocols for multiplex PCR, single-base primer extension, and generation of mass spectra as per the manufacturer’s instructions (Sequenom, San Diego, CA). Genotyping was conducted at the Functional Genomics Core, Children’s Hospital Oakland Research Institute. Four multiplexed assays were done using each 5 ng genomic DNA. Blanks and duplicates were included in the plates.

**Genetic Data Quality Control**

Systematic quality control (QC) was applied to all genotypes. The per individual call rate had to be higher than 70% to be included in the analyses. SNPs found to be out of Hardy-Weinberg (p<10^-5^) or missing more than 30% of genotypes were removed from the dataset. After QC, 666 individuals remained, 24 of which were eliminated because they were not born in the island and had no Puerto Rican ancestors. The final sample included 642 participants with information for 93 AIMs. The median call rates were 0.989 for the remaining individuals and 0.963 for the AIMs. A final list of the SNPs used is included in Table S1. This set of markers showed a difference in allele frequency (δ) of 0.606 between Africans and Native Americans, 0.449 between Africans and Europeans, and 0.383 between Europeans and Native Americans.

**Admixture estimates**

Ancestral reference groups were represented by data on the same AIM panel on West African, European, and Native American populations. West African samples included 37 individuals recruited in London, UK, and South Carolina, USA, and were kindly provided by Paul McKeigue. The European ancestrals consisted of 42 European American samples from Coriell’s North American Caucasian panel. The Native American samples included 30 individuals (15 Mayans and 15 Nahuas, from villages in remote areas of Mexico) provided by Mark Shriver.

Individual ancestral estimates (IAE) were calculated using the Bayesian methods implemented in the program STRUCTURE [5,6]. We assumed an admixture model with unlinked markers and with 20,000 burn-ins and 20,000 further iterations. We used three ancestral populations (K=3), African, European and Native American, and included genotype data on the ancestral populations previously described. After preliminary calculations, we detected five samples among the ancestral individuals that showed a substantial proportion (> 10%) of admixture from other ancestral groups and were eliminated from further calculations. We tested several models using different number of generations since the admixture event took place, and we concluded that 15 generations gave the best results (the smallest lnP(D)).

**Geographic and historic variables**

We located each individual by the latitude and longitude of the centroid of each census block. Overall, our 642 individuals with data on their genetic ancestry fell within 128 different census blocks across the island (see Table S2 for a list of census blocks and Figure 1A for a distribution map). Distribution of 1990 census blocks was obtained from the U.S. Census Bureau ([http://www.census.gov](http://www.census.gov/)) and longitude and latitude coordinates were obtained using ArcView GIS 9.3.1 software (ESRI, Redlands, California, USA). For some analyses, we grouped individuals based on municipality and region. Regions were defined geographically in six groups: East, West, Center, North, South, and Metro (see Figure S1A). The Metropolitan region pooled together the municipalities of San Juan, Caguas, Guaynabo, and Bayamón. These municipalities are in the urban agglomeration area of San Juan, include almost a quarter of the island population and cannot be considered representative of their geographic area, but of a separate entity. For each census block, elevation from mean sea level was obtained from NASA’s Shuttle Radar Topography Mission (<http://www2.jpl.nasa.gov/srtm/>) (Table S2). We obtained the coordinates of Puerto Rico from ArcView and calculated great circle distances in kilometers to the closest coastal point.

One of the goals of this study was to establish the historical elements that have contributed to the current distribution of admixture across Puerto Rico. Six ports were analyzed for their role in the African slave trade to Puerto Rico. San Juan was the only legally authorized port to introduce slaves to Puerto Rico from 1513 (initial granting to import slaves to Puerto Rico) to 1812. In 1812, five additional ports were authorized to import African slaves: Aguadilla, Cabo Rojo, Fajardo, Mayagüez, and Ponce [7]. Municipalities containing sugar mills during the 16th century were compiled from Gelpí Baíz (2000) [8]: Añasco, Bayamón, Canóvanas, Loíza, Toa Alta, Toa Baja, and Yabucoa. The coordinates of the centroid of each municipality were established using the ArcView software. Great circle distances to the closest port and to the closest municipality with sugar mills were calculated for every individual in the dataset. Ports and sugar mills location have been plotted in Figure 1A.

We also collected the sugarcane plantation area and the production of sugar and molasses per district in 1830 compiled by de Córdoba (1831) [9] (see Table S3 and Figure S1B). For each individual in the sample, we calculated a weighted value for all three variables (sugarcane area, sugar and molasses production) using the following equation:

where *S_i_* is the plantation-related weighted variable (sugarcane area or sugar or molasses production) for individual *i*, *d_ik_* is the great circle distance of individual *i* to the centroid of region *k*, *S_k_* is the plantation-related variable for district *k*, and N is 7, the number of historical districts with sugar plantations (Table S3). The weighted variables for sugar and sugarcane were almost completely correlated (ρ = 0.95, p < 10^-4^), so only the weighted variables for sugar and molasses were included in the analyses.

**Geographic and historical analyses**

Geostatistical methods were used to analyze the spatial pattern of variation in admixture. Interpolation contour maps based on the underlying pattern of spatial correlation were performed using the estimation method Kriging [10]. Interpolative methods are based on spatial proximity, assuming that an unknown data point would be more similar to sampled data points that are geographically closer than to sampled points further away. Most common types of interpolation predict data values at unsampled locations by weighting the observed data points inversely proportional to their geographic distance from the location (or using different exponentials of the weighting, i.e. inverse distance squared). Compared to these ad-hoc weighting methods, Kriging uses a weighting based on a theoretical distribution inferred from the spatial variability inherent in the data (as a review, check [11]). In the present study, we used MapViewer 6 software with 50 nodes to construct interpolation plots of admixture across Puerto Rico. The final plots were constructed using a linear variogram model. Exponential and gaussian variogram models were also tested and the resulting plots retrieved similar patterns of spatial distribution to the ones shown in the present manuscript.

The independence of admixture estimates from neighboring individuals was tested by means of spatial autocorrelation. The dependency of the data assumes that estimates for geographically close individuals are more highly related than those for distant individuals. In this study, we used Moran’s I and Getis and Ord’s G statistics [12,13] to detect these correlations using ArcView. Moran’s I function evaluates whether the admixture pattern expressed is clustered, dispersed, or random, and calculates a Z score to evaluate if the clustering or dispersion is statistically significant. Getis and Ord’s G statistic measures concentrations of high or low values for a study area, and also calculates a Z score to evaluate if the G value is the result of random chance or statistically significant. Both statistics were calculated by using inverse Euclidean and inverse Euclidean squared distances between pairs of individuals. Given the small size of the island the difference between Euclidean distance and Great Circle distance is negligible.

Multiple linear regression models were implemented to assess the relationships between genetic ancestry and different geographical and historical variables. The variables that we tested included elevation from mean sea level, distance to the coast, distance to ports used during the African slave trade, distance to the location of sugar mills, and sugar and molasses weighted production (see Geographic and historic variables section and Table S3). Three models were built: one for the whole island, one for the East region, and one for the other 5 regions combined. Variables included in the final models were selected using a backwards deletion stepwise regression and only main effects (no interaction terms) were considered. Bootstrap analyses were performed 10,000 times to test the robustness of findings and to check for bias. These models were run in R 2.10.0 (R Project for Statistical Computing, <http://www.r-project.org/>).

Additionally, we used geographically weighted regression (GWR) models to assess the accuracy of the final regression across the geography of Puerto Rico (at the individual level). GWR allows the modeling of processes that vary over space and results in a set of local parameter estimates which can be mapped and combined with interpolation to show the variation of a parameter across the study region [14]. Compared to a global regression model, GWR allows local parameters to be estimated using the following general model:

y_i_ = β_0_(u_i_,v_i_) + Σ_k_ β_k_(u_i_,v_i_)x_ik_ + ε_i_,

where y is the dependent variable (ancestry) for individual i, β is the parameter being estimated for the independent variables (geographical elements), (u_i_,v_i_) are the geographic coordinates of individual i, and ε is a measure of error. We run these models to estimate the individual R^2^ values for each sample using the spgwr package in R 2.10.0.

**Socioeconomic Status (SES)**

Information relevant to socioeconomic status (SES) was acquired from all participants, such as educational level, neighborhood, house description and the occupation of the subject or the household’s main provider. To minimize inconsistencies, the SES of all subjects was determined by a single investigator as an overall estimation based on the above information. SES was grouped in five ordinal categories: low (L), medium low (ML), medium (M), medium high (MH), and high (H). For some analyses, these categories were numerically recoded from 1 to 5. SES is notoriously difficult to measure. In order to test the reliability of our estimations, we compared them to an independent and more thorough collection of SES variables available only for 496 of the 800 original individuals in the sample. This information was collected through an independent study led by Dr. Luis Avilés of the Dept. of Social Sciences of the University of Puerto Rico at Mayagüez. The correlation between both assessments was very high (ρ > 0.75), so the original SES estimation, which was available in all the individuals, was determined to be appropriate and was used in the analyses.

SES differences at the regional and municipal level were analyzed by ANOVA and Pearson’s chi-square tests. Spatial autocorrelation analyses using Moran’s I and Getis and Ord’s G statistics were performed to analyze the independence of SES from neighboring individuals in a similar fashion to the spatial autocorrelation tests previously described for admixture estimates. Ordinal logistic regression (OLR) models were used to estimate the proportion of variation in SES across individuals that could be explained by geography and ancestry. The geographic variables tested included geographic region (Central, East, Metro, North, South, and West), elevation from mean sea level (in meters), and distance to the nearest coast (in km). African ancestry was the ancestral contribution included in the models because it showed the highest correlation coefficient with SES. Interaction terms were also tested to disentangle the independent and joint effects. Variables and interactions included in the final models were selected using a backwards deletion stepwise regression. These models were run using the *lrm()* function in the Design packages in R 2.10.0 (R Project for Statistical Computing). Given the small number of individuals with high SES (only 12 individuals), the medium high and high (4 and 5) SES categories were merged for OLR analyses to increase the small cell size and minimize numerical instability. This model uses the parallel regression assumption that the difference between any two outcome groups is the same. Because of this assumption, odds ratios (OR) and 95% confidence intervals (95% CI) for every independent variable were calculated for increases in SES from 1 to 2-4, from 1-2 to 3-4, or from 1-3 to 4. We performed 10,000 bootstraps at the individual level to test the robustness of findings.

REFERENCES

1. Martinez-Cruzado JC, Toro-Labrador G, Viera-Vera J, Rivera-Vega MY, Startek J, et al. (2005) Reconstructing the population history of Puerto Rico by means of mtDNA phylogeographic analysis. Am J Phys Anthropol 128: 131-155.

2. Martinez-Cruzado JC, Toro-Labrador G, Ho-Fung V, Estevez-Montero MA, Lobaina-Manzanet A, et al. (2001) Mitochondrial DNA analysis reveals substantial Native American ancestry in Puerto Rico. Hum Biol 73: 491-511.

3. Yaeger R, Avila-Bront A, Abdul K, Nolan PC, Grann VR, et al. (2008) Comparing genetic ancestry and self-described race in african americans born in the United States and in Africa. Cancer Epidemiol Biomarkers Prev 17: 1329-1338.

4. Tsai HJ, Choudhry S, Naqvi M, Rodriguez-Cintron W, Burchard EG, et al. (2005) Comparison of three methods to estimate genetic ancestry and control for stratification in genetic association studies among admixed populations. Hum Genet 118: 424-433.

5. Falush D, Stephens M, Pritchard JK (2003) Inference of population structure using multilocus genotype data: linked loci and correlated allele frequencies. Genetics 164: 1567-1587.

6. Pritchard JK, Stephens M, Donnelly P (2000) Inference of population structure using multilocus genotype data. Genetics 155: 945-959.

7. Díaz Soler LM (2005) Historia de la esclavitud negra en Puerto Rico. [San Juan]: Editorial de la Universidad de Puerto Rico. 439 p., [435] leaves of plates p.

8. Gelpí Baíz E (2000) Siglo en blanco : estudio de la economia azucarera en el Puerto Rico del siglo XVI (1540-1612). San Juan, P.R.: Editorial de la Universidad de Puerto Rico. xviii, 414 p. p.

9. de Córdoba PT (1831) Memorias geográficas, históricas, económicas y estadísticas de la isla de Puerto Rico. San Juan: Oficina del Gobierno.

10. Isaaks EH, Srivastava RM (1989) Applied geostatistics. New York: Oxford University Press. xix, 561 p. p.

11. Relethford JH (2008) Geostatistics and spatial analysis in biological anthropology. Am J Phys Anthropol 136: 1-10.

12. Moran PAP (1948) The interpretation of statistical maps. J R Stat Soc B 10: 243-251.

13. Getis A, Ord K (1992) The analysis of of spatial association by use of distance statistics. Geogr Anal 24: 189-206.

14. Fotheringham AS, Brunsdon C, Charlton M (2002) Geographically weighted regression : the analysis of spatially varying relationships. Chichester, England ; Hoboken, NJ, USA: Wiley. xii, 269 p. p.
